# Controlling evanescent waves using silicon photonic all-dielectric metamaterials for dense integration

**DOI:** 10.1038/s41467-018-04276-8

**Published:** 2018-05-14

**Authors:** Saman Jahani, Sangsik Kim, Jonathan Atkinson, Justin C. Wirth, Farid Kalhor, Abdullah Al Noman, Ward D. Newman, Prashant Shekhar, Kyunghun Han, Vien Van, Raymond G. DeCorby, Lukas Chrostowski, Minghao Qi, Zubin Jacob

**Affiliations:** 1 Department of Electrical and Computer Engineering, University of Alberta, Edmonton, AB T6G 1H9 Canada; 2 School of Electrical and Computer Engineering and Birck Nanotechnology Center, Purdue University, West Lafayette, IN 47907 USA; 3 Department of Electrical and Computer Engineering, Texas Tech University, Lubbock, TX 79409 USA; 4 Department of Electrical and Computer Engineering, University of British Columbia, Vancouver, BC V6T 1Z4 Canada; 5 Shanghai Institute of Microsystem and Information Technology, Chinese Academy of Sciences, Shanghai, 200050 China

## Abstract

Ultra-compact, densely integrated optical components manufactured on a CMOS-foundry platform are highly desirable for optical information processing and electronic-photonic co-integration. However, the large spatial extent of evanescent waves arising from nanoscale confinement, ubiquitous in silicon photonic devices, causes significant cross-talk and scattering loss. Here, we demonstrate that anisotropic all-dielectric metamaterials open a new degree of freedom in total internal reflection to shorten the decay length of evanescent waves. We experimentally show the reduction of cross-talk by greater than 30 times and the bending loss by greater than 3 times in densely integrated, ultra-compact photonic circuit blocks. Our prototype all-dielectric metamaterial-waveguide achieves a low propagation loss of approximately 3.7±1.0 dB/cm, comparable to those of silicon strip waveguides. Our approach marks a departure from interference-based confinement as in photonic crystals or slot waveguides, which utilize nanoscale field enhancement. Its ability to suppress evanescent waves without substantially increasing the propagation loss shall pave the way for all-dielectric metamaterial-based dense integration.

## Abstract (web summary)

Miniaturization of optical components could give way to dense photonic-integrated circuits. Here, the authors demonstrate the control of evanescent waves using all-dielectric metamaterials and show that they can reduce cross-talk and bending loss, which limit the integration density in photonic circuits.

## Introduction

One of the long-standing goals of nanophotonics is the integration of electronic and photonic circuitry on a single CMOS chip for applications ranging from information processing and data centers to massively parallel sensing^1–11^. This necessarily requires miniaturization with low power consumption in optical interconnects, active as well as passive photonic devices. There are two major figures of merit in designing photonic devices for a densely integrated circuit. One is the cross-talk, which occurs due to the field overlap of two adjacent photonic waveguides, and the second is the radiation loss at sharp bends which limits the integration density^6^.

**Fig. 1:**
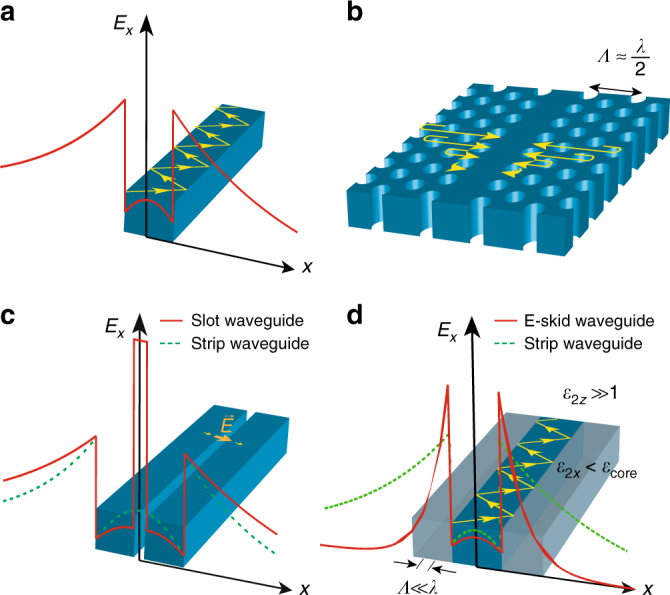
Fundamental differences between dielectric waveguides on an SOI platform. **a** Strip waveguide; **b** photonic crystal waveguide; **c** slot waveguide; **d** e-skid waveguide. The skin-depth in the cladding of e-skid waveguide is shorter compared to the other structures due to the strong effective anisotropy of the multilayer cladding. This counterintuitive approach can have a cladding with a higher average index than the core and marks a departure from interference-based confinement as in photonic crystal waveguides or slot waveguides which utilize nanoscale field enhancement

Plasmonic waveguides can strongly reduce cross-talk and bending loss owing to the sub-diffraction nature of light coupling to the free electrons of metals^12–19^. However, the large ohmic loss of metals restricts the application of plasmonic structures for photonic integration^20,21^. Over the last decade, many efforts have been made to miniaturize photonic components using all-dielectric structures^22–36^. Figure 1 illustrates a few classes of dielectric waveguides for light confinement in photonic chips. Strip waveguides, the most common type of waveguides for routing light in a silicon chip, are composed of a silicon channel surrounded by silicon oxide^6^ (Fig. 1a). Owing to the high contrast between the refractive index of the core and the cladding, light is confined inside the core as a result of total internal reflection (TIR). However, the mode size is seen to increase as we reduce the core size which hampers the use of strip waveguides to further miniaturize photonic circuits^37^. Photonic crystal waveguides can confine light inside a line defect due to Bragg reflection^40^ (Fig. 1b). These waveguides perform efficiently at very sharp bends^41^, however, the integration density is limited as the periodicity of Bragg reflectors is on the order of the wavelength and it cannot be perturbed by another waveguide nearby^42^. Additionally, slot waveguides have been proposed to confine light inside a sub-wavelength low-index gap surrounded by high-index dielectric rods^43^ (Fig. 1c). To satisfy the continuity of the normal component of the displacement current at the high-contrast interface, the electric field peaks inside the gap, leading to light confinement but at the cost of skin-depth expansion in the cladding. This causes cross-talk between adjacent waveguides and radiation loss at sharp bends in dense photonic-integrated circuits.

Several alternative approaches inspired from atomic physics^44,45^, transformation optics^46^, and inverse design algorithms^47,48^ have also been proposed to minimize the cross-talk and the bending loss. However, these techniques add design complexity and often require modification of the core as well as cladding^46^. They are not implementable on a large-scale and cause propagation loss fundamentally limiting device performance. Thus, a new low-loss and scalable platform is needed for CMOS-foundry compatible dense photonic integration with low cross-talk and reduced bending loss.

In this paper, we demonstrate a platform that is fundamentally different from the existing approaches for designing CMOS compatible, ultra-compact, and low-loss waveguides using all-dielectric anisotropic metamaterials. Our approach works based on photonic skin-depth engineering of evanescent waves in the cladding using a recently proposed degree of freedom in TIR^37,49^. To describe the light confinement mechanism in our waveguides, first, we experimentally demonstrate the phenomenon of relaxed TIR in anisotropic metamaterials. These relaxed conditions allow the control of evanescent wave decay, which is the fundamental origin of cross-talk and bending loss in silicon photonic devices. As illustrated in Fig. 1d, we use these anisotropic metamaterials as a cladding for on-chip dielectric waveguides fabricated on a monolithic silicon-on-insulator (SOI) platform. As a result, cross-talk is reduced down to −30 dB in the photonic circuit. Furthermore, we use a transformation optics approach to show that the radiation loss at sharp bends is strongly influenced by the skin-depth in the cladding. We experimentally show that the anisotropic metamaterial cladding can simultaneously reduce the bending loss at sharp bends up to three times compared with conventional silicon strip waveguides. We clarify the counterintuitive nature of light confinement in our approach compared with existing photonic crystal, slot waveguide, and graded index waveguide methods^38,43,50–53^. Our work shows that all-dielectric anisotropy on-chip presents a scalable route to simultaneously improve cross-talk and bending loss with propagation loss as low as ≈3.67 dB/cm. For completeness, we show the improvements in figures of merit of our achieved platform with recent state-of-the-art photonic designs (see Table 1).

**Table 1:** Performance comparison between an extreme skin-depth (e-skid waveguide) and other dielectric waveguides

Reference	Cross-talk	Propagation loss
Superlattice^44^	−20 dB	>20 dB/cm
Adiabatic elimination^45^	−21.9 dB	N/A
Inverse design^48^	−22.9 dB	>300 dB/cm
Dissimilar waveguides^78^	−20 dB	N/A
Sinusoidal waveguides^79^	−26.8 dB	>600 dB/cm
This work (e-skid)	−30 dB	3.67 dB/cm

It is seen that the cross-talk is significantly reduced in e-skid waveguides at the negligible cost of propagation loss in comparison with other approaches

## Results

### Relaxed TIR

We demonstrate that only a single component of the dielectric tensor governs TIR in anisotropic media opening a new degree of freedom to control evanescent waves. In conventional TIR, if *n*_1_>*n*_2_ (*n*_1_ and *n*_2_ are the refractive index of the medium 1 and medium 2, respectively) and the incident angle is greater than the critical angle ($\theta_{\rm{c}} = \mathop {{\mathrm {sin}}}\nolimits^{ - 1} \left( {{{n}}_2/{{n}}_1} \right)$), light is reflected back to the first medium and decays evanescently in the second medium. However, we have recently found that the TIR conditions at the interface of an isotropic and an anisotropic dielectric are relaxed to^37,54^:

$$n_1 > \sqrt {\varepsilon _{2x}} ,\;p\;{\mathrm{polarization}}$$

1 $$n_1 > \sqrt {\varepsilon _{2y}} ,\;s\;{\mathrm{polarization}}$$

where $\left[ {\varepsilon _{2x}\varepsilon _{2y}\varepsilon _{2z}} \right]$ is the permittivity tensor of the second medium, and the interface between the two media lies on the *yz* plane. As a result, the critical angle for *s* and *p* polarizations differ:

$$\theta_{\rm{c}} = \mathop{{\mathrm {sin}}}\nolimits^{-1} \left({\sqrt {\varepsilon_{2x}}/{n}_1} \right),\;p\;{\mathrm{polarization}}$$

2 $$\theta_{\rm{c}} = \mathop{{\mathrm {sin}}}\nolimits^{-1} \left({\sqrt {\varepsilon _{2y}} /n_1} \right),\;s\;{\mathrm{polarization}}$$

We emphasize that these relaxed conditions for *p* polarized incident light allow us to arbitrarily increase or decrease the permittivity in the *z* direction, whereas still preserving TIR. This hitherto un-utilized degree of freedom can thus be used to control the skin-depth of evanescent waves. If $\varepsilon _{2z} \gg 1$, evanescent waves decay faster than in vacuum allowing for strong light confinement inside dielectric waveguides^37,49^. In this limit, note that the averaged index in anisotropic medium 2 can be larger than the refractive index in isotropic medium 1 yet the light will be totally reflected above the critical angle.

**Fig. 2:**
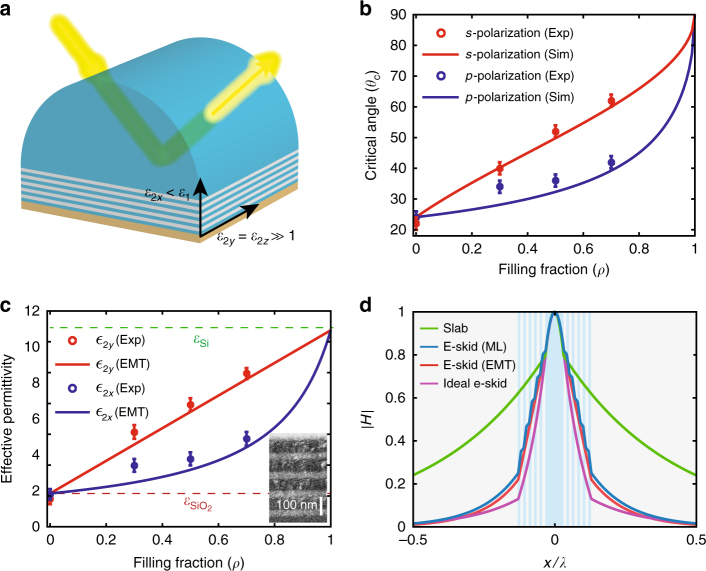
Relaxed total internal reflection. **a** Schematic of the relaxed TIR experiment. We measure the reflection from the Si prism and Si/SiO_2_ multilayer interface for both *s* and *p* polarizations at *λ*=1550 nm. Multiple samples with different *ρ* have been fabricated. The periodicity for all samples is 100 nm and the total thickness of the multilayer is 500 nm. A 200 nm layer of tungsten is deposited on top of the multilayer (brown) to suppress the reflection from the air interface. **b** The measured critical angle for *s* and *p* polarizations vs. the silicon filling fraction in comparison with effective medium theory (EMT) calculations. Error bars represent the instrument limit of the measuring device. In contrast with the conventional phenomenon of TIR, observable differences between the critical angles is seen for different polarizations in agreement with relaxed TIR theory. **c** Retrieved effective permittivity of the multilayer from the critical angle measurements show strong anisotropy in agreement with EMT. The refractive index of Si and SiO_2_ are taken to be 3.4 and 1.47, respectively, for the theoretical calculations. The inset shows the SEM image of an Si/SiO_2_ multilayer with *ρ* = 0.5. **d** Normalized calculated magnetic field profile for the TM mode of a conventional slab waveguide compared to extreme skin-depth (e-skid) waveguides with Si/SiO_2_ multilayer cladding. The blue and gray regions represent Si and SiO_2_, respectively. The core size, *Λ*, and *ρ* are 100 nm, 30 nm, and 0.5, respectively. The multilayer anisotropic claddings strongly affect the decay of the evanescent wave in the cladding. The practical multilayer structure performs close to an ideal anisotropic case ($\varepsilon _{2x} = \varepsilon _{{\mathrm{SiO}}_2}$ and $\varepsilon _{2z} = \varepsilon _{{\mathrm{Si}}}$)

We implement this anisotropy using Si/SiO_2_ multilayers with subwavelength thicknesses (Fig. 2a) at the telecommunication wavelength (*λ* = 1550 nm). Figure 2 highlights the contrast between relaxed TIR and conventional TIR, showing the measured light reflection at the interface of a hemi-cylindrical Si prism and the anisotropic multilayer metamaterial. The periodicity of the multilayer is *Λ* = 100 nm and five periods have been deposited on the prism. The Maxwell–Garnett effective medium theory (EMT)^55^ predicts that the multilayer with subwavelength periodicity demonstrates strong anisotropy^37,56–59^ at the operating wavelength ($\varepsilon _{2y} = \varepsilon _{2z} = \varepsilon _{{\mathrm{Si}}}\rho + \varepsilon _{{\mathrm{SiO}}_2}(1 - \rho )$ and $1/\varepsilon _{2x} = \rho /\varepsilon _{{\mathrm{Si}}} + (1 - \rho )/\varepsilon _{{\mathrm{SiO}}_2}$ where *ρ* is the fill fraction of silicon). Note we are in the effective medium metamaterial limit $\left( {\Lambda \ll \lambda } \right)$ away from the photonic crystal regime $\left( {\Lambda \sim \lambda } \right)$. Reflection occurs on two interfaces, the primary silicon-metamaterial interface and the secondary metamaterial-air interface. We have deposited a thin tungsten layer on top of the multilayer metamaterial to attenuate the unwanted reflection from the secondary metamaterial/air interface. Figure 2b displays different measured critical angles for *s* and *p* polarizations. Various samples with different fill fractions have been fabricated. As *ρ* deviates from 0 and 1, the multilayer displays effective anisotropy and the critical angle for *s* and *p* polarized incidences are separated in agreement with relaxed TIR theory in Eq. (2). The effective permittivity of the multilayer structure (extracted from the critical angle) shows strong anisotropy and is in good agreement with EMT (Fig. 2c). It is clearly seen that the critical angle depends on only one component of the permittivity tensor for each polarization irrespective of the fill fraction. With the extra degree of freedom afforded by the other components of the dielectric tensor, this anisotropic structure can be used as a cladding for conventional dielectric waveguides to control the evanescent waves and skin-depth in the cladding^37,60^. Figure 2d displays the effect of the multilayer anisotropic cladding on reducing of the evanescent wave skin-depth as compared to a conventional slab waveguide. Note that this evanescent wave engineering approach in extreme skin-depth (e-skid) waveguides is owing to the fast variation of the evanescent fields in a subwavelength high-index-contrast superlattice (Fig. 2d). This is fundamentally different from other light confinement strategies which function on interference of propagating waves (photonic crystals) or field enhancement in a slot^38,43,51–53^.

### On-chip e-skid waveguides

**Fig. 3:**
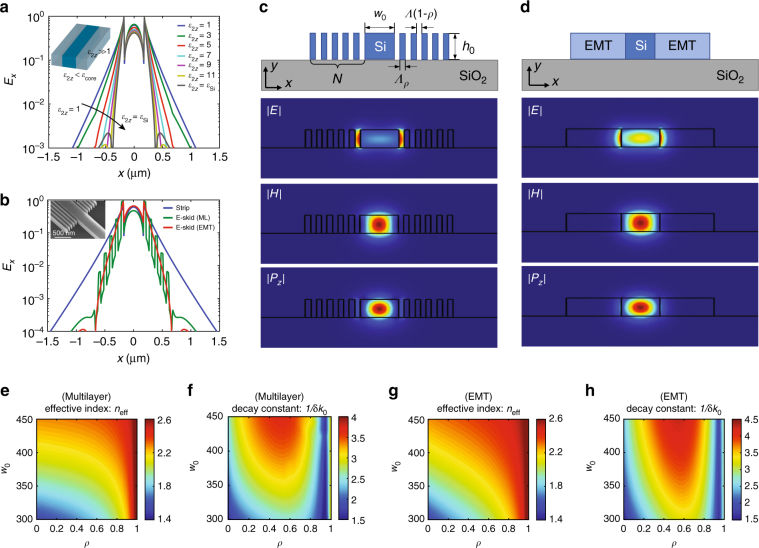
On-chip extreme skin-depth (e-skid) waveguides. **a** Ideal on-chip e-skid waveguide; light is confined by total internal refection inside the core and as the effective anisotropy of the multilayer metamaterial cladding is increased, evanescent waves of TE-like modes decay faster in the cladding in comparison with the field in strip waveguides ($\varepsilon _{2z} = 1$). Note that $\varepsilon _{2x} = 1$ for all cases. **b** The simulated electric field profile at the center of the e-skid waveguide with multilayer (green) and homogenized metamaterial ($\varepsilon _{2x} = 1.85$ and $\varepsilon _{2z} = 6.8$) (red) claddings, in comparison with a strip waveguide (blue). Inset shows the SEM image of the fabricated e-skid to strip waveguide transition. **c**, **d** Schematic and field profiles of **c** realistic e-skid waveguide with multilayer claddings and **d** its equivalent model with EMT claddings. **e**, **f** Effective refractive indices $\left( {n_{{\mathrm{eff}}} = k_z \hskip -3.5pt \prime /k_0} \right)$ and **g**, **h** normalized decay constants ($k_x \hskip -2.5pt {\prime\prime} /k_0 = 1/\delta k_0$) of the e-skid waveguide as functions of the core width *w*_0_ and filling fraction *ρ*: with **e**, **g** multilayer and **f**, **h** EMT claddings, respectively. Geometric parameters are *h*_0_ = 220 nm, *w*_0_=350 nm, *Λ* = 100 nm, *ρ* = 0.5, and *N* = 5, unless otherwise indicated. The free space wavelength is *λ*=1550 nm. Simulations confirm that the cladding achieves effective all-dielectric anisotropy as well as the increased decay constant of evanescent waves outside the core

To impact the field of silicon photonics, the one-dimensional e-skid waveguide (Fig. 2d) has to be implemented on an SOI platform. This places stringent restrictions on monolithic fabrication, minimum feature sizes, and deviations in field profiles due to quasi-2D behavior. Our design to adapt relaxed TIR and skin-depth engineering $\left( {\delta \ll \lambda } \right)$ on-chip is shown in Fig. 3. Here the conventional quasi-2D strip waveguide is surrounded by a cladding made of alternating vacuum-silicon sub-wavelength ridges which achieves strong anisotropy ($\varepsilon _{2x} < \varepsilon _{{\mathrm{core}}}$ and $\varepsilon _{2z} \gg 1$). Our goal is to confine the TE-like mode (its dominant electric field component is *E*_*x*_) used in conventional silicon strip waveguides. Since the TM-like mode is polarized in the *y* direction, the electric field does not probe/feel the anisotropy of the cladding. Hence, the metamaterial cladding does not play any role in TM mode confinement. For more details about the polarization effect, see Supplementary Figures 6 and 7.

Figure 3a emphasizes the counterintuitive nature of the confinement caused by anisotropic media which beats the confinement of conventional high index-contrast interface consisting of silicon surrounded by vacuum (blue curve in Fig. 3a). As it is seen in Fig. 3a, if we increase the index of the cladding only in the *z* direction, whereas the index in the other directions are fixed, the evanescent wave decays faster in the cladding and the skin-depth reduces drastically. Note, the average cladding index increases but the mode decay length decreases. This behavior is in fundamental contrast to confinement in conventional strip waveguides with isotropic claddings or graded index claddings^53^ where decreasing the contrast between the core index and cladding index results in slow decay of evanescent waves, not faster decay^49^.

Figure 3b shows the field profile of a practical e-skid waveguide with the multilayer metamaterial cladding $\left( {\mathit{\Lambda} \ll \mathit{\lambda} } \right)$. We can see the practical multilayer cladding shows strong effective anisotropy to ensure the fast decay of the evanescent wave in the cladding. The 2D field profiles in a plane perpendicular to the propagation direction and comparison of the practical e-skid waveguide with a homogeneous anisotropic cladding are shown in Figs. 3c and 3d. A good agreement with the effective medium approximation is seen. Figures 3e-h illustrate the effective modal index and the decay constant in the cladding for a wide range of core sizes and the fill fractions of silicon in the multilayer cladding. First, a strong agreement is seen between EMT and practical multilayer structures, indicating that the cladding indeed achieves effective all-dielectric anisotropy. Second, the effective modal index is below the core index indicative of relaxed TIR as the mechanism of confinement. As expected, simulations prove that the primary role of the on-chip multilayer cladding is to increase the decay constant of evanescent waves $\left( {k_x \hskip -3.5pt {\prime \prime }/k_0} \right)$, as shown in Figs. 3f, h, and it does not significantly change the effective modal index of propagating waves $\left( {k_z \hskip -3.5pt \prime /k_0} \right)$, as shown in Figs. 3e, g. It is seen that the largest decay constant is achieved at $\rho \approx 0.5$ where the strongest anisotropy is attained. Note that *ρ* = 0 corresponds to the case of no cladding. We also emphasize that the power in the core is not compromised by the presence of the skin-depth engineering cladding and increased anisotropy causes an enhancement in power confinement.

This light confinement strategy in e-skid waveguides is fundamentally different from that in photonic crystal waveguides. Our multilayer structures function in the deep subwavelength limit $\left( {\Lambda \ll \lambda } \right)$, not the photonic crystal limit $\left( {\Lambda \sim \lambda } \right)$ and the performance of the e-skid waveguide is independent of the multilayer periodicity or disorder (see Supplementary note 2). Furthermore, there are no Dyakonov wave solutions in e-skid waveguides as the optical axis of the anisotropic cladding is perpendicular to the interface and the direction of propagation^22,61–65^. It is also important to note that the main goal of the anisotropic multilayer cladding is to control the decay constant of evanescent waves on-chip, not to achieve birefringence in the effective modal index of propagating waves as achieved previously in sub-wavelength grating structures^26,50,51,66^, multi-slotted waveguides^52^, or nonlinear phase matching applications^67^.

### Cross-talk in e-skid waveguides

The skin-depth of evanescent waves is the fundamental origin of cross-talk between waveguides that hinders dense photonic integration. Cross-talk, or power coupling between photonic devices, arises owing to the perturbation of the optical mode when the evanescent tail of one waveguide overlaps with a nearby waveguide. However, if we control the skin-depth in the cladding, we can reduce the perturbation due to the adjacent waveguide, subsequently reducing the cross-talk to surpass the integration limit in current silicon photonics.

**Fig. 4:**
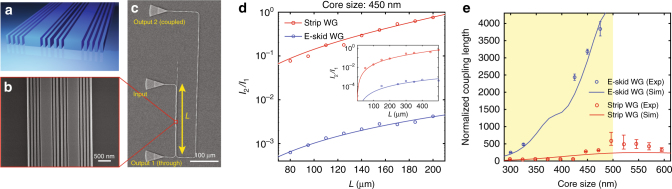
Cross-talk in e-skid waveguides. **a** Schematic of coupled e-skid waveguides on an SOI platform. The waveguide height, center-to-center separation between the two waveguides (*s*), and *Λ* are 220 nm, 1000 nm, and 120 nm, respectively. A cladding oxide is also added on the top of the waveguides. The number of ridges between the waveguides is dictated by the waveguide core size. **b** Top view SEM image of the coupled e-skid waveguides. **c** The experimental setup to measure the cross-talk between the two waveguides at the telecommunication wavelength (*λ*=1550 nm). Light is in-coupled to the first waveguide through the middle grating coupler. The second waveguide is coupled to the first waveguide for a length of *L*. In this experiment, the bending radius is 5 μm, hence, we can ignore the bending loss. **d** The ratio between the measured output powers for strip waveguide and e-skid waveguide vs. *L* at the telecommunication wavelength. The ratio for the e-skid waveguide is two orders of magnitude lower, indicating that far less power is coupled to the second waveguide. The inset shows the ratio for the waveguides without the top cladding oxide. In this case, the metamaterial cladding can increase the coupling length up to 30 times or reduce the cross-talk -30 dB. See Supplementary Figure 8 for more details. **e** Comparison of the simulated and measured coupling length for e-skid waveguides and strip waveguides. The coupling length is normalized to the wavelength. Error bars represent the standard deviation of the fitting curves. The optimum match between the simulation and experiment is achieved when *ρ* = 0.6 for the cladding of e-skid waveguides. The coupling length for e-skid waveguides is an order of magnitude larger in comparison with strip waveguides (shaded region). The coupling length for strip waveguides with larger core size decreases because the overlap between the evanescent tails is increased although more power is confined inside the core (unshaded region)

We now demonstrate this drastic reduction in the cross-talk made possible with the anisotropy in the cladding (Fig. 4a). The top view image of coupled e-skid waveguides fabricated on an SOI chip is shown in Fig. 4b. The power exchanged between two identical lossless coupled waveguides is:

$$I_1 = I_0\mathop {{\mathrm {cos}}}\nolimits^2 \left( {\frac{\pi }{{2L_{\mathrm{c}}}}L} \right),$$

3 $$I_2 = I_0\mathop{{\mathrm {sin}}}\nolimits^2 \left( {\frac{\pi }{{2L_{\mathrm{c}}}}L} \right),$$

where *I*_0_ is the input power, *L* is the distance for which the two waveguides are coupled, and *L*_c_ is the coupling length. Note *L*_c_ is defined such that if the waveguides are coupled for a distance *L*=*L*_c_, complete power transfer occurs from the first waveguide to the adjacent waveguide. Figure 4c illustrates the experimental setup to measure the coupling length. Light is in-coupled to the first waveguide through the grating coupler^68^ and the second waveguide is coupled to the first waveguide over a distance of *L*. The center-to-center separation between the two waveguides is only 1000 nm. The output power of the two waveguides is out-coupled by the two grating couplers at the ends of each waveguide. Note that we can ignore the propagation loss in this experiment as the total length does not exceed a few hundred microns, whereas the propagation loss is 3.67 dB/cm at the operating wavelength *λ*=1550 nm (see Supplementary Note 6 for details about the characterization of the propagation loss). The ratio between the measured powers for strip waveguides and e-skid waveguides are shown in Fig. 4d. This highlights that the analytical expression $\left({I_2/I_1 = \mathop{{\mathrm {tan}}}\nolimits^2 \left( {\pi L/2L_{\mathrm{c}}} \right)} \right)$ matches the experimental data. It is clearly seen that the coupled power to the second waveguide is almost two orders of magnitude lower for the e-skid waveguide. The measured and simulated^69^ coupling length normalized to the wavelength for different core sizes is plotted in Fig. 4e for waveguides with the same separation distance of 1000 nm. It is seen that the coupling length for the TE-like mode of e-skid waveguides is an order of magnitude higher than that for strip waveguides allowing miniaturization of photonic-integrated circuits without considerable cross-talk between the waveguides. Note that increasing the core size in conventional strip waveguides, which corresponds to higher power confinement inside the core, does not guarantee the reduction in cross-talk (unshaded region Fig. 4e) as the evanescent tails are not controlled. These drastically increased *L*_c_ are due to the reduced skin-depth with a higher anisotropy, reducing the waveguide cross-talk significantly in comparison with other dielectric structures, which have been proposed to reduce the cross-talk (Table 1). Note that as the TM-like mode has a negligible electric field component in the *x* direction, it does not probe/feel the dielectric anisotropy of the cladding. As expected, this results in higher cross-talk for TM-modes compared with strip waveguides (see Supplementary Figures 6 and 7 for more details about the polarization effect and the proposed design to control the skin-depth of the TM-like mode).

### Bending loss in e-skid waveguides

**Fig. 5:**
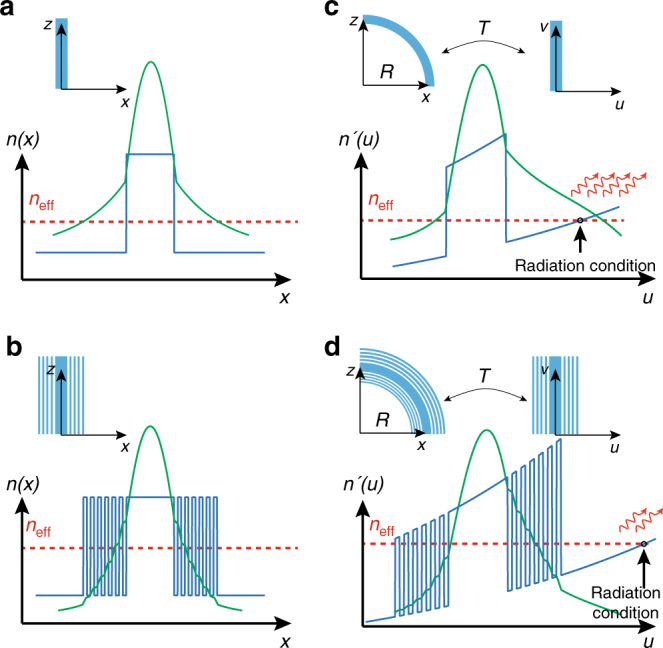
Curved waveguides and skin-depth engineering. **a**, **b** Refractive index profile (blue) and the magnetic field profile (green) of **a** a straight strip waveguide and **b** a straight e-skid waveguide. **c** The corresponding bent waveguides **c**, **d** can be transformed to straight waveguides with inhomogeneous refractive index profiles in a new coordinate system. If the local refractive index of the cladding exceeds the effective modal index $\left( {n{\prime}(u_0) > n_{{\mathrm{e}}ff}} \right)$, the waveguide mode starts radiating. This occurs at the radiation condition point denoted by black arrows. The multilayer metamaterial cladding in e-skid waveguides suppresses the evanescent wave field beyond this radiation condition point. Thus, in comparison with conventional bent waveguides, lesser power is radiated owing to curvature effects

The critical phenomenon of bending loss^70–72^ can also be reduced by adding the anisotropic metamaterial cladding. The counterintuitive connection of bending loss and skin-depth is revealed by an approach adapted from transformation optics^70,73^. Here, we consider a curved waveguide in the *xz* plane with a 90° bend and a bending radius of *R* $\left( {R \gg \delta } \right)$. The center of the curved waveguide is at the origin (Figs. 5b, d (inset)). If we apply the transformation: $u + iw = R{\mathrm l}{\mathrm n}\frac{{x + iz}}{R},\;v = y$^70,73^, the curved waveguide is mapped to a straight waveguide in the *uw* plane (Figs. 5b, d). This causes the refractive index of the transformed waveguide to become inhomogeneous in the new coordinate system as^70,73^: $n{\prime}\left( u \right) = n\left( {x(u)} \right){\mathrm e}^{u/R}$, where $n\left( {x\left( u \right)} \right)$ is the refractive index of the straight waveguide as shown in Figs. 5a, b. Bending loss or radiative power leakage from the core occurs in this straight inhomogeneous index waveguide when the local index of the cladding exceeds the effective modal index (*n*_eff_). Hence, if we suppress the field near and beyond this radiation condition point (Figs. 5b, d), we can reduce the bending loss. Note that due to the spatial transformation of coordinates, the electromagnetic fields are also transformed causing the expansion of the skin-depth (*δ*) on the right-hand side of the waveguide and shrinkage of it on the left-hand side (Figs. 5b, d):

$$\delta _{{\mathrm{right}}} \approx \delta + 2\delta ^2/R,$$

4 $$\delta _{{\mathrm{left}}} \approx \delta - 2\delta ^2/R.$$

However, if we add the anisotropic metamaterial cladding, we can reduce the skin-depth. As a result, less power will be radiated at the radiation condition point, leading to reduced bending loss.

**Fig. 6:**
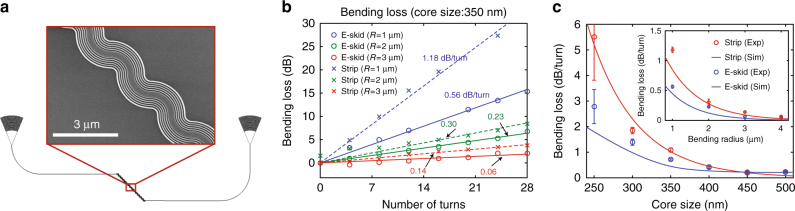
Bending loss in e-skid waveguides. **a** Layout and zoomed-in SEM images of the test devices to characterize the bending loss. **b** Measured bending losses with different number of turns; circles and crosses are the e-skid and strip waveguides, respectively, and blue, green, and red are at different bending radiuses of *R* = 1, 2, and 3 μm. Each experimental result is fitted with a linear line: e-skid (solid) and strip (dashed) lines. The core size is $w_0 = 350$ nm. **c** Characterized bending loss vs. core size ($R = 1 {\mathrm{{ \mu} }}$m): e-skid (red) and strip (blue) waveguides. Inset shows the characterized bending loss vs. bending radius ($w_0 = 350$ nm). Red and blue lines are the simulated bending losses of the e-skid and strip waveguides, respectively. Error bars represent the standard deviation of the fitting curves. Other parameters ($h_0,\Lambda ,\rho ,N$, and *λ*) are the same as in Fig. 3. Confinement of the evanescent waves in e-skid waveguides due to the metamaterial cladding helps to reduce the bending loss at sharp bends in photonic-integrated circuits. This effect is stronger for waveguides with smaller core sizes, as a considerable amount of the power lies outside the core and decays slowly

To confirm the effect of the anisotropic metamaterial claddings on the bending loss, we have investigated the bending losses of the e-skid and strip waveguides, both experimentally and numerically^74^. To characterize the bending losses, we have cascaded 90° bent waveguides sequentially with different numbers of turns (Fig. 6a and Supplementary note 4), then characterized the bending loss per turn by comparing the transmissions. Figures 6b, c shows the measured bending losses vs. the core size and the bending radius. In all cases, the bending losses with the e-skid waveguide is lower than that with the strip waveguide, which is due to the reduced skin-depth with the anisotropic metamaterial claddings. Note that if the fabrication advancements on the CMOS platform allowed us to reduce the feature size and approach an ideal anisotropic cladding $\left( {\Lambda \to 0} \right)$, we would achieve further reduction of the bending loss for TE-like modes in e-skid waveguides (See Supplementary note 4 for more details as well as the bending loss calculation for TM-like modes). Our simulations account for all sources of bending loss including radiation and mode-mismatch.

## Discussion

In summary, we have introduced a photonic platform that can add the critical but overlooked functionality of controlling evanescent waves to the CMOS foundry. We have shown that high-index contrast grating structures in the deep sub-wavelength limit can act as an all-dielectric metamaterial cladding for simultaneously achieving TIR and controlling the skin-depth of evanescent waves. The coupling length is improved more than an order of magnitude and the bending loss is improved three times compared with conventional on-chip waveguides with an average propagation loss of 3.67 dB/cm at telecommunication wavelengths. The decreased photonic skin-depth regime has not been realized till date but has been attempted in initial studies^75^. This is because an anisotropic all-dielectric metamaterial response requires more than one period of the unit cell to control evanescent wave decay^37,49,54^. Although we use electron-beam lithography (EBL) as a convenient prototyping technique for the sub-wavelength structures, these devices can in principle be fabricated using deep ultraviolet CMOS foundries; specifically, advanced 193 nm immersion lithography technology has been used to fabricate silicon photonic devices with feature sizes down to 50 nm^76^. Our work paves the way for all-dielectric metamaterials to enter the practical realm of CMOS-foundry photonics to achieve improved photonic-integrated circuits.

## Methods

### Relaxed total internal reflection

Si/SiO_2_ multilayers were fabricated using magnetron sputtering and magnetron reactive sputtering, respectively. Silicon and silica were both deposited at a power of 150 W using a pulsed power supply at a frequency of 150 kHz and off time of 0.5 μs for silicon and 0.8 μs for silica. A special substrate holder was built to hold the prisms during deposition. With the new substrate holder and the large thickness of the prism, the film properties would change as the substrate was much closer to the target, producing films with higher loss. Reducing the deposition power produced lower loss films. A 200 nm thick layer of tungsten was deposited at 300 W, 150 kHz, and 0.5 μs on top of the multilayer structure at each fill fraction. The prism was illuminated with a 1530 nm narrow line width laser. A broadband linear polarizer placed in the beam path created *s* and *p* polarized light. The incident angle was increased in increments of 2° from 10° to 80°. A Newport Optics Optical Power Meter calibrated to a wavelength of 1530 nm was then used to measure the reflected power. The two reflections at the prism/air interface in the optical path were accounted for when comparing experiment with EMT simulations.

### On-chip platform 1

The on-chip devices for measuring cross-talk in the main text and bending loss in Supplementary Figure 9 were fabricated using a JEOL JBX-6300FS EBL system^77^ operated at 100 keV energy, 8 nA beam current, and 500 μm exposure field size. A silicon-on-insulator wafer (220 nm thick silicon on 3 μm thick silicon dioxide) has been used. A solvent rinse and hot-plate dehydration baked. Then, hydrogen silsesquioxane resist (HSQ, Dow-Corning XP-1541-006) was spin-coated at 4000 rpm, and then hot-plate baked at 80 °C for 4 min Shape placement by the machine grid, the beam stepping grid, and the spacing between dwell points during the shape writing, were 1 nm, 6 nm, and 6 nm, respectively. An exposure dose of 2800 μC/cm^2^ was used. The resist was developed by immersion in 25% tetramethylammonium hydroxide for 4 min., followed by a flowing deionized water rinse for 60 s, an isopropanol rinse for 10 s. Then blown dry with nitrogen. The inductively coupled plasma etching in an Oxford Plasmalab System 100 was used to remove silicon from unexposed areas, with a chlorine gas flow of 20 sccm, ICP power of 800 W, pressure of 12 mT, bias power of 40 W, and a platen temperature of 20 °C, resulting in a bias voltage of 185 V. During etching, perfluoropolyether vacuum oil was used to mount chips on a 100 mm silicon carrier wafer. Cladding oxide was deposited using plasma enhanced chemical vapor deposition in an Oxford Plasmalab System 100 with nitrous oxide flow of 1000.0 sccm, a silane flow of 13.0 sccm, high-purity nitrogen flow of 500.0 sccm, high-frequency RF power of 120 W, pressure at 1400 mT, and a platen temperature of 350 °C. Chips rest directly on a silicon carrier wafer during deposition and are buffered by silicon pieces on all sides to aid uniformity.

To characterize the on-chip devices, a custom-built automated test setup was used^6^. An Agilent 81635A optical power sensor was used as the output detector and Agilent 81600B tunable laser as the input source. The wavelength was swept in 10 pm steps from 1500 to 1600 nm. To maintain the polarization state of the light, a polarization maintaining fiber was used for coupling the TE polarization into the grating couplers^68^. A polarization maintaining fiber array was used to couple light in/out of the chip.

### On-chip platform 2

For the bending loss experiment in the main text, the cross-talk experiment in Supplementary Figure 8, the insertion loss experiments in Supplementary Figure 13, and the propagation loss experiment in Supplementary Figure 14, a standard SOI wafer is used as a substrate with 2 μm buried oxide and 220 nm top silicon layer. Diluted HSQ with methyl isobutyl ketone was spun on the substrate as a negative-tone electron-beam resist layer. The resist layer was exposed by a 100 kV EBL system, VB6-UHR (Raith), which is capable of 2 nm beam step resolution. After the development of the resist, the top silicon layer was etched by Cl_2_/O_2_ based reactive-ion plasma etching tool (Panasonic P610) to transfer the waveguide pattern from the resist to the silicon layer.

### Data availability

The data that support the findings of this study are available from the corresponding authors upon request. Some examples are available at https://github.com/lukasc-ubc/SiEPIC_EBeam_PDK/tree/master/Examples/eskid.

## Electronic supplementary material

Supplementary Information
