# Supplementary material for: Controlling evanescent waves using silicon photonic all-dielectric metamaterials for dense integration
Source: Nat Commun. 2018 May 14;9:1893. doi: 10.1038/s41467-018-04276-8 (PMC5951946; doi:10.1038/s41467-018-04276-8)
Supplement: Supplementary file 1 — Supplementary Information [file 41467_2018_4276_MOESM1_ESM.pdf]

Supplementary information: controlling evanescent waves using silicon-photonic all-dielectric metamaterials for dense integration

Saman Jahani[1,2,*] Sangsik Kim[2,3,*] Jonathan Atkinson[1], Justin C. Wirth[2], Farid Kalhor[1,2], Abdullah Al Noman[2], Ward D. Newman[1,2], Prashant Shekhar[1], Kyunghun Han[2], Vien Van[1], Raymond G. DeCorby[1], Lukas Chrostowski[4], Minghao Qi[2,5,†] and Zubin Jacob[1,2‡]

[1]*Department of Electrical and Computer Engineering, University of Alberta, Edmonton, AB T6G 1H9 Canada.*

[2]*School of Electrical and Computer Engineering and Birck Nanotechnology Center, Purdue University, West Lafayette, IN 47907 USA.*

[3]*Department of Electrical and Computer Engineering, Texas Tech University, Lubbock, TX 79409*

[4]*Department of Electrical and Computer Engineering, University of British Columbia, Vancouver, BC, V6T 1Z4 Canada.* and

[5]*Shanghai Institute of Microsystem and Information Technology, Chinese Academy of Sciences, Shanghai 200050, China.*

SUPPLEMENTRY NOTE 1. RELAXED TOTAL INTERNAL REFLECTION

Supplementary Figure 1 displays the field profile for p-polarized plane-wave propagation through an Si/SiO_2 multilayer sandwiched between two Si half spaces. The incident angle is $\theta = 10°$ (top) and $\theta = 80°$ (bottom). In both cases, $\Lambda = 100$ nm and $\rho = 0.5$, and light is incident from the left side at $\lambda = 1550$ nm. The gray and yellow regions represent Si and SiO_2 layers, respectively. The critical angle of the effective medium is $\theta_c = 32.33°$ based on relaxed-TIR theory. It is seen that above the critical angle, the electromagnetic wave is totally reflected back to the left side and forms a standing wave on the left side but decays inside the multilayer and negligible power is transmitted to the right half space. However, when the incident angle is below the critical angle, most of the power is transmitted through the multilayer because the total thickness of the slab is thin in comparison with the wavelength.

SUPPLEMENTRY NOTE 2. EFFECT OF PERIODICITY AND DISORDER ON E-SKID WAVEGUIDES

Although practical metamaterial structures for realization of strong effective anisotropy are usually periodic, the effective macroscopic electromagnetic response of metamaterials does not stem from the periodicity (or unit-cell size). As long as the periodicity of the metamaterial building blocks is deeply subwavelength, the change in the periodicity does not change the electromagnetic response of metamaterials. Indeed, according to the effective medium theory (EMT), which has been explained in the main text, the effective constitutive parameters of multilayer metamaterials depend on the permittivity of the building blocks and their filling fraction, not the periodicity of the multilayer.

To demonstrate the effect of periodicity on the performance of e-skid waveguides, we analyzed 1D e-skid waveguides with multilayer claddings of the same total size, but different periodicities as illustrated in Supplementary Fig. 2. We also analyzed a case where the multilayer is not periodic. We can see that since the effective permittivity for all three cases is the same, the performance of the waveguides are almost identical. This demonstrates that the light confinement mechanism in e-skid waveguides and photonic crystal waveguides are fundamentally different. In photonic crystal waveguides, the periodicity is a requirement and a change in periodicity strongly affects the performance of the waveguide.

We have also studied the effect of periodicity on the modal effective index (n_{eff}) of TE-like and TM-like modes of a single on-chip e-skid waveguide as a function of core size at λ=1550 nm. Supplementary Figure 3 shows that EMT is an extremely accurate approximation to homogenize the cladding when the unit cell size is considerably smaller than the operating wavelength. The effect of the unit-cell size is more obvious on the effective index of the TE-like mode because the field intensity is higher in the cladding region for these modes.

SUPPLEMENTRY NOTE 3. CROSS-TALK

In the main text, we have reported the coupled power at a single wavelength. However, the coupling between two waveguides is a function of frequency in general. Supplementary Figure 4 displays the spectral response of strip and e-skid couplers. Since multilayer metamaterials are non-resonant broadband structures, multilayers show strong anisotropy for a wide range of wavelengths as long as the period is considerably smaller than the wavelength. Hence, as shown in Supplementary Fig. 4, the multilayer cladding causes the cross-talk to decrease for a broad range of frequencies.

* These authors contributed equally to this work

† mqi@purdue.edu

‡ zjacob@purdue.edu

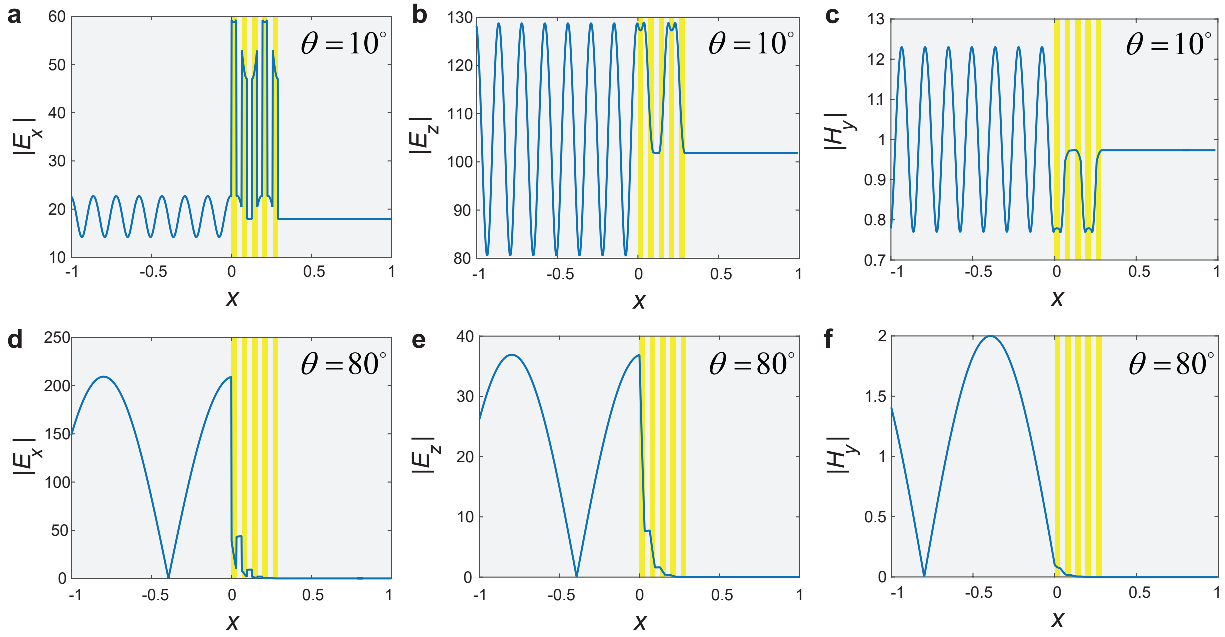

Supplementary Figure 1. Comparison of the field profile for the p-polarized electromagnetic wave propagation through an Si/SiO_2 multilayer anisotropic metamaterial below (**a-c**) and above (**d-f**) the critical angle ($\theta_c = 32.33°$). For both cases, $\Lambda = 100$ nm and $\rho = 0.5$. It is seen that incident light is totally reflected although the total thickness of the metamaterial layer ($t = 500$ nm) is subwavelength ($\lambda = 1550$ nm).

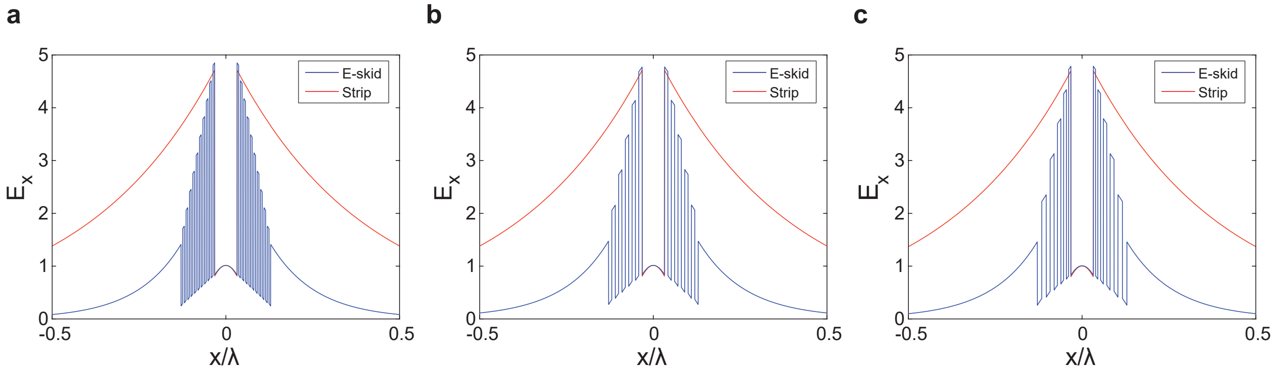

Supplementary Figure 2. **Effect of the cladding periodicity on 1D e-skid waveguides.** The comparison of the x-component of the electric field at λ=1550 nm. The core is silicon with a size of 100 nm. The cladding is Si/SiO_2 multilayer with $\rho = 0.5$. (a) The periodicity is Λ=10 nm. (b) The periodicity is Λ=30 nm. (c) The periodicity linearly varies from Λ=20 nm to Λ=45 nm. The total thickness of the waveguide in all cases is 400 nm. The modal effective index (n_{eff}) for the three cases are 1.9062, 1.8864, and 1.8864, respectively. The ratio between the power confined inside the core and total power (η) for the three cases are 30.98%, 29.79%, and 29.99%, respectively. n_{eff}= 1.5281 and η= 12.36% when the cladding is removed. It is clearly seen that as long as the periodicity is subwavelength, the performance of e-skid waveguide depends on the effective permittivity of the multilayer cladding not the periodicity or disorder in the cladding. Thus the waveguiding mechanism in e-skid waveguides is fundamentally different from that in photonic crystal waveguides.

An important challenge of alternative waveguides is mode conversion efficiency from the grating coupler to the waveguide. The comparison of the output level of the through waveguide shows that the coupling efficiency from the grating coupler to the e-skid waveguides is very close to the coupling efficiency in strip waveguides. Moreover, as we explain in Supplementary Note 5, the mode conversion efficiency from a strip waveguide to an e-skid waveguide with the same core size is above 98%. This means that e-skid waveguides can easily be integrated to the silicon photonics chip. Supplementary Figure 5 illustrates the normalized coupling length versus the center-to-center separation for coupled e-skid and strip waveguides. For a compact photonic circuit, minimum separa-

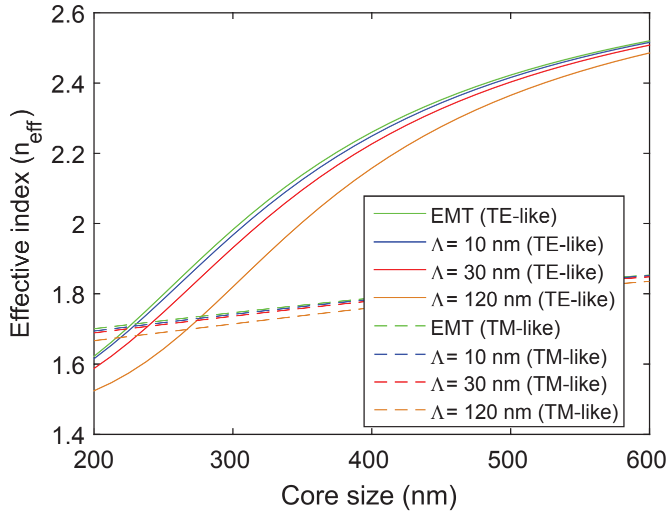

Supplementary Figure 3. **Effect of the cladding periodicity on the modal index.** Effect of the multilayer cladding periodicity (Λ) on the modal effective index of the TE-like and TM-like modes of a single e-skid waveguides on SOI platform. The simulation parameters are the same as those in Fig. 4 in the main text. The effective permittivity of the cladding is $\begin{bmatrix} \varepsilon_{2x} & \varepsilon_{2y} & \varepsilon_{2z} \end{bmatrix} = \begin{bmatrix} 2.33 & 7.6 & 7.6 \end{bmatrix}$ which is calculated using effective medium theory (EMT). As $\Lambda \to 0$, there is an excellent match between EMT and multilayer simulations.

tion with maximum decoupling between adjacent waveguides are required [1]. However, as it is seen in Supplementary Fig. 5, the coupling length exponentially decreases as the separation is reduced. This trade-off limits the integration density of photonic circuits. Due to the reduced skin-depth in e-skid waveguides, we can reduce the decoupled separation further and increase the integration density of photonic integrated circuits. We should emphasize that the decoupled separation in slot waveguides and photonic crystal waveguides are even more than that in strip waveguides [1].

We also contrast the effect of anisotropy on TE-like and TM-like modes of on-chip e-skid waveguides. Supplementary Figure 6 shows the coupling length for TE-like and TM-like modes versus ε_z while ε_x is fixed in the cladding. As the anisotropy increases, we can reduce the skin-depth in the cladding for TE-like mode. Thus, the coupling length between the two coupled waveguides is strongly enhanced because the overlap between evanescent waves of adjacent waveguides is suppressed. On the other hand, since the electric field in the x direction is negligible for TM-like modes, these modes cannot feel the anisotropy of the cladding. Hence, the anisotropic cladding cannot help to control the momentum of evanescent waves for TM-like modes.

To control the evanescent waves of TM-like modes, the cladding must be anisotropic in the yz plane and it must be added beneath or above the waveguide core. Supplementary Figure 7a (inset) illustrates a simplified structure to control the skin-depth of TM-like modes. We keep the permittivity in the y direction fixed, but we change it in the z direction to control the anisotropy. As we increase the permittivity in the z direction, the optical mode is confined inside the core and the overlap between the evanescent tales is reduced. Hence, as shown in Supplementary Fig. 7a, the coupling length increases. Supplementary Figure 7b displays a practical implementation of the anisotropic cladding for the TM-like modes composed of Si/SiO_2 multilayer claddings. In this case, Λ = 40 nm and the silicon filling fraction is $\rho = 0.5$. Supplementary Figure 7c and 7d show the normalized electric field profile of the even mode of the coupled strip and e-skid waveguides, respectively. It is seen that the skin-depth of the evanescent waves in e-skid waveguides is reduced, and as a result, the coupling length has been increased from 14.5λ for strip waveguides case to 30.6λ for e-skid waveguides case. Note that if we add the multilayer on top of the waveguide core as well, more confinement is achievable and the cross-talk can be reduced further.

Cross-talk in E-skid Waveguides Without an Upper Cladding

In an e-skid waveguide, the waveguide cross-talk can be reduced further by increasing the anisotropy of the metamaterial. Supplementary Figure 8a shows the schematic layout to measure the cross-talk between coupled e-skid waveguides, and Supplementary Fig. 8b shows an SEM image of coupled e-skid waveguides. The parameters for the e-skid waveguide are set to $h_0 = 220$ nm, $\Lambda = 100$ nm, $\rho = 0.5$, and $N = 5$; this sets the separation distance between the two waveguides (edge-to-edge) to be $d_{\text{sep}} = 550$ nm, which is less than a free space wavelength ($\lambda = 1550$ nm). In this case, there is no upper SiO_2 cladding and the cross-section is similar to that in Fig. 3c of the main manuscript (Si/air multilayers). This leads to a higher anisotropy in metamaterials and reduces the skin-depth of the guided mode compared to the case of Si/SiO_2 multilayers. To evaluate the waveguide cross-talk, as in the main text, we have characterized the coupling length L_c by measuring the output power ratio (I_2/I_1) for different lengths (L) of devices. Red and blue circles in Supplementary Figs. 8c-8e are the measured output power ratio for e-skid and strip waveguides, respectively, and dashed lines are their respective fitting curves with $I_2/I_1 = \tan^2(\pi L/2L_c)$. Supplementary Figures 8c-8e are with different core widths of w_0 =350, 400, and 450 nm, respectively. Supplementary Figure 8f summarizes the normalized coupling lengths (L_c/λ) that are characterized through Supplementary Figs. 8c-8e: e-skid (red circles) and strip (blue circles) waveguides. The solid lines are their respective simulation results that match well with the experimental measurements. Notice that, in every case, the coupling length of an e-skid waveguide is much longer than that of a strip waveguide. The maximum coupling length of more than 3 cm is achieved

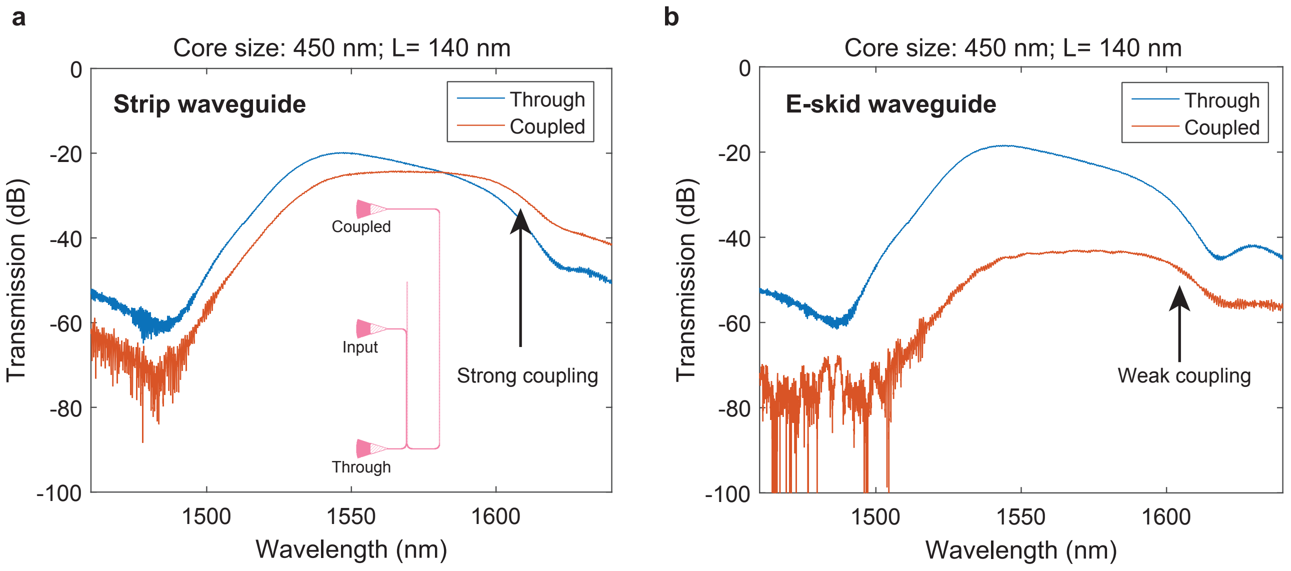

Supplementary Figure 4. **Measured transmission spectra to the through and coupled waveguides. a,** Strip waveguide; **b,** e-skid waveguide. (Inset) Schematic representation of the experimental setup to measure the coupling length. Light is in-coupled through the middle grating coupler. The waveguide core size and the coupling distance (L) is 450 nm and 140 nm, respectively, in both cases. The other parameters of the waveguides are the same as the waveguides in Fig. 4 in the main text. The anisotropic cladding causes coupling to the second waveguide to drop almost 20 dB. This helps to reduce the cross-talk in dense photonic integrated circuits.

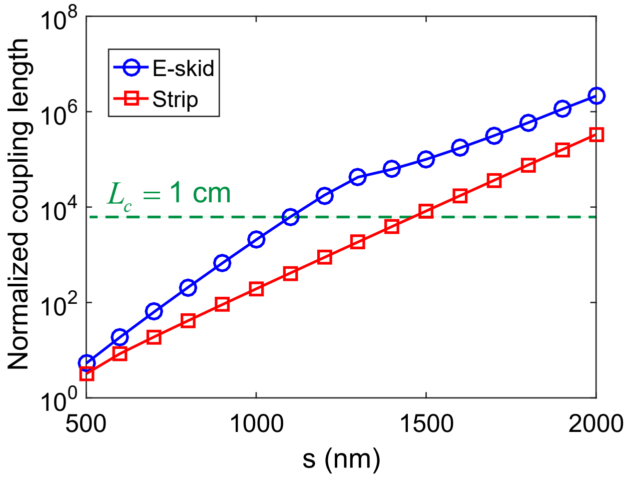

Supplementary Figure 5. **Normalized coupling length (L_c/λ) versus the center-to-center separation between the coupled waveguides (s).** $w_0 = 450$ nm, $\Lambda = 100$ nm, and $\rho = 0.5$. Other simulation parameters are the same as those in Fig. 3 in the main text. $L_c = 1$ cm has been defined as the minimum decoupling length. In this case, the minimal separation length (center-to-center) in e-skid waveguides and strip waveguides are 1104 nm and 1466 nm, respectively. This shows that e-skid waveguides will have higher performance for dense photonic integration than strip waveguides.

($w_0 = 450$ nm), and, when w_0 is 400 or 450 nm, the coupling length for an e-skid waveguide is about 30 times longer in comparison with strip waveguide.

SUPPLEMENTRY NOTE 4. BENDING LOSS

We have also simulated [2] and measured the bending loss when the waveguides are coated by an SiO_2 upper cladding (Supplementary Fig. 9). An oxide cladding is usually used to protect the device and to integrate it with electronic interconnects [3]. In this experiment, the input power is divided into two paths with equal lengths. It is seen that the bending loss is considerably reduced due to the metamaterial cladding even when the core size is large.

In the main text, we have presented the magnetic field of curved strip waveguides and e-skid waveguides using a transformation optics approach to demonstrate the effect of skin-depth on the bending loss. However, for accurate calculation of bending loss, 3D full-wave simulations are required. Supplementary Figure 10 shows the top view of the simulated [2] magnetic field profile of TE-like mode for curved strip and e-skid waveguides. It is seen that for all-cases the skin-depth extends on the right side and is compressed on the left side in agreement with the transformation optics calculations. The performance of e-skid waveguides improves if we approach the effective medium theory limit ($\Lambda \to 0$) (Supplementary Fig. 10). As we mentioned earlier, the TM-like mode of on-chip waveguides does not feel the anisotropy of the cladding because the electric field in the x direction is negligible. Hence, the confinement of the TM-like mode in e-skid cladding is the same as that in an isotropic cladding as shown in Supplementary Fig. 11.

We compare the bending loss for both TE-like and TM-

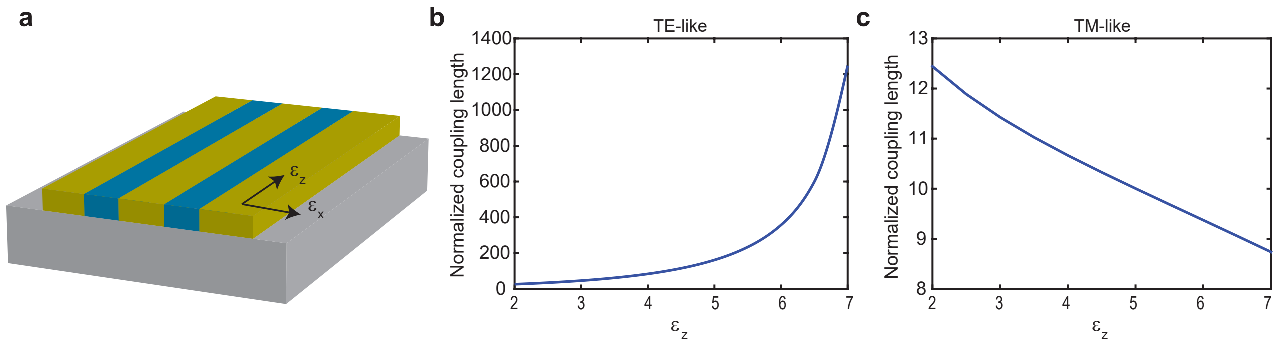

Supplementary Figure 6. **Effect of cladding anisotropy on the coupling length for TE-like and TM-like modes of e-skid waveguides. a,** Schematic representation of the two coupled Si waveguides with a core size of 300 nm, height of 220 nm, and center-to-center separation of 1000 nm operating at $\lambda = 1550$ nm. The waveguides have been covered by a homogeneous anisotropic metamaterial (yellow) with $\varepsilon_x = 2$ and $\varepsilon_y = \varepsilon_z$. **b,** Coupling length for TE-like mode versus ε_z. **c,** Coupling length for TM-like mode versus ε_z. As we enhance the anisotropy of the cladding, the coupling length of TE-like mode increases as a result of skin-depth reduction in the cladding.

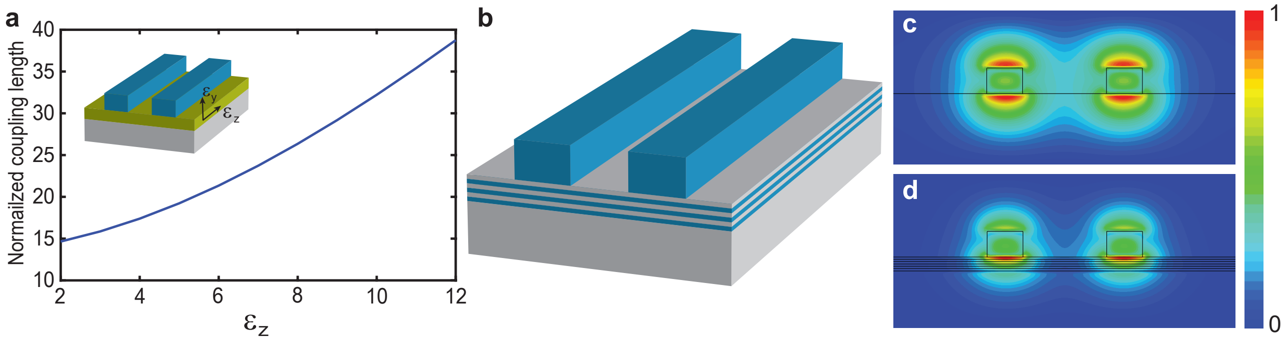

Supplementary Figure 7. **E-skid waveguides for controlling the evanescent waves of the TM-like mode. a,** The schematic representation of proposed e-skid waveguides for controlling the skin-depth of the TM-like mode (inset). Since the electric field of the TM-like mode is polarized in the y direction, the cladding must be anisotropic in yz plane. To simplify the structure, the anisotropic cladding (yellow) is implemented only underneath the waveguides. The plot shows the normalized coupling length between the two waveguides versus the anisotropy of the metamaterial layer while the permittivity in the y direction is fixed to be 2.2. The thickness of the layer is 120 nm. The waveguide parameters are the same as that in Supplementary Fig. 6. **b,** Practical Si/SiO_2 multilayer structure to confine the evanescent waves and reduce the cross-talk between the two waveguides. $\Lambda = 40$ nm and the silicon filling fraction is $\rho = 0.5$. **c** and **d,** The normalized electric field profile of the even mode of the coupled strip and e-skid waveguides, respectively. It is seen that the multilayer structure helps to confine the mode inside the waveguide and reduces the overlap between the evanescent waves of the two waveguides. This results in the increase of the coupling length from 14.5λ to 30.6λ.

like modes of e-skid and strip waveguides in Supplementary Fig. 12. As we mentioned in the main text, the additional degree of freedom in total internal reflection can be used to control evanescent waves of p-polarized (TE-like) modes. Hence, the anisotropic cladding helps to reduce the bending loss of the TE-like mode in e-skid waveguides in comparison with strip waveguides. However, this degree of freedom does not exist for s polarized (TM-like) modes. Since the effective permittivity of the cladding for the TM-like mode is larger than the permittivity of SiO_2, the evanescent waves decay slower for TM-like modes in e-skid waveguides. Thus, the skin-depth extends more and more power is radiated at sharp bends in comparison with strip waveguides.

SUPPLEMENTRY NOTE 5. MODE CONVERSION EFFICIENCY

The mode conversion efficiency between the e-skid waveguide and the strip waveguide are also investigated. For the mode conversion efficiency, we use a racetrack resonator (Supplementary Fig. 13) to characterize the insertion loss efficiency [4]. Supplementary Figure 13c shows the normalized transmission spectra for the e-skid (red) and strip (blue) waveguides. The resonances are slightly shifted with metamaterials due to the different propagation phase in the e-skid waveguide (see Fig. 3 in the main text), but the extinction ratios are almost similar suggesting a similar cavity loss. Supplementary Figures 13d and 13e are the fitted resonances for the strip

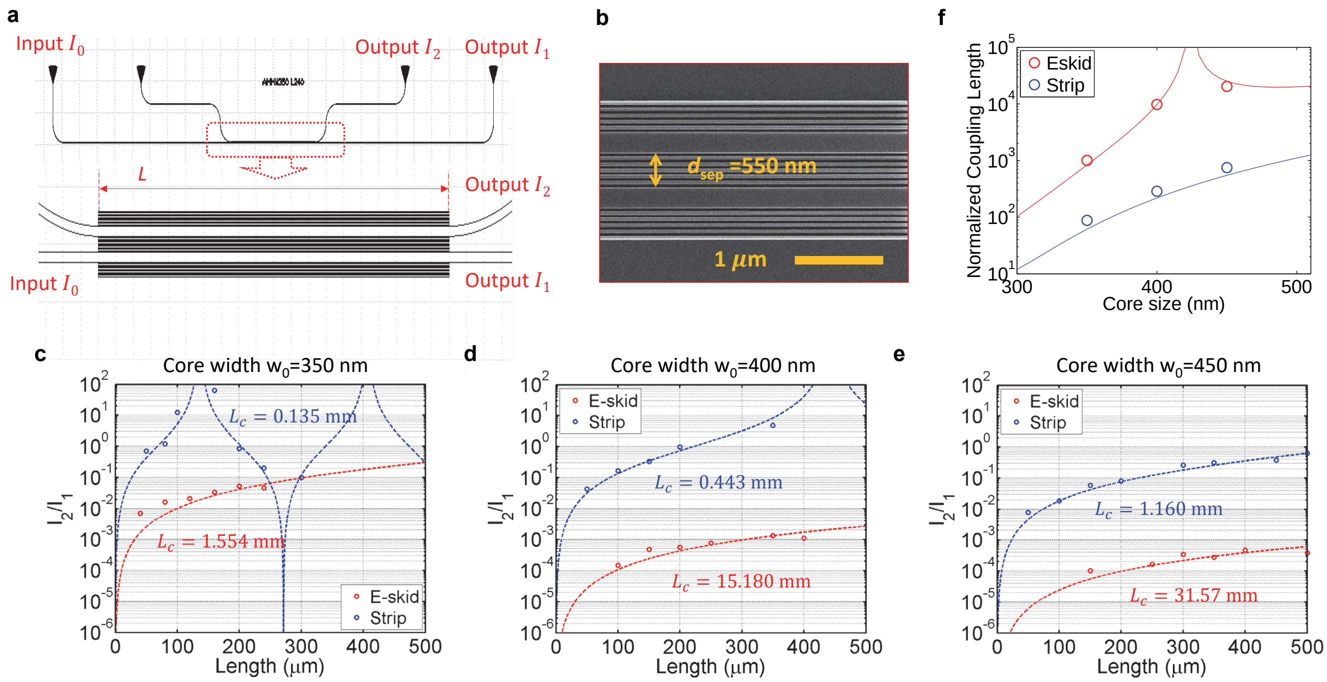

Supplementary Figure 8. **Cross-talk in e-skid waveguides without an Upper cladding.** **a,** Schematic layout to measure the cross-talk and **b,** SEM image of coupled e-skid waveguides on an SOI platform. Geometric parameters are set to $h_0 = 220$ nm, $\Lambda = 100$ nm, $\rho = 0.5$, and $N = 5$, setting the separation distance between the two waveguides to be $d_{\text{sep}} = 550$ nm. There is no upper cladding. **c-e,** Coupling length L_c characterization by measuring the output power ratio (I_2/I_1) vs. device length L; core widths w_0 are (**c**) 350 nm, (**d**) 400 nm, and (**e**) 450 nm, respectively. Red and blue circles are the measured power ratio for the e-skid and strip waveguides, respectively, and dashed lines are their fitting curves with $I_2/I_1 = \tan^2(\pi L/2L_c)$. **f,** Normalized coupling length (L_c/λ) for the e-skid (red circles) and strip (blue circles) waveguides. Red and blue lines are their respective simulation results.

and e-skid waveguides, respectively, and the intrinsic quality factors are 26,518 and 24,535, which correspond to the round-trip losses of 0.6893 dB/round and 0.7466 dB/round, respectively [5]. Since we have four interfaces for the mode conversion, a rough estimation of the mode conversion loss is about 0.0143 dB/facet, which corresponds to a mode conversion efficiency of 99.6%. The mode conversion efficiencies (CEs) with other resonances are also characterized and plotted in Supplementary Fig. 13c. This high mode conversion efficiency between the strip waveguide and the e-skid waveguide in TE-like modes indicates a low insertion loss, and suggests a high compatibility with the previously used PIC devices.

SUPPLEMENTRY NOTE 6. PROPAGATION LOSS

To characterize the propagation losses in the e-skid and the strip waveguides, we have fabricated two sets of waveguides (e-skid and strip waveguides) with different lengths of the straight section. The waveguide parameters are set to $w_0 = 450$ nm, $h_0 = 220$ nm, $w_\rho = w_{(1-\rho)} = 50$ nm, and $N = 5$; then the lengths of the straight section of the e-skid and strip waveguides are varied from 0 to 1.8 mm. Supplementary Figure 14 shows the measured transmission powers of e-skid (blue circles) and strip (red circles) waveguides at $\lambda_0 = 1550$ nm, for different device lengths. Solid lines in each figure are the linear fitting curves that give the propagation losses of 2.65 dB/cm and 3.43 dB/cm to strip and e-skid waveguides, respectively. The propagation losses for strip and e-skid waveguides at different wavelengths have also been characterized. The average losses for strip and e-skid waveguides for wavelengths between 1540 nm to 1560 nm are 1.84 dB/cm and 3.67 dB/cm, respectively, with a standard deviation of 1.4 dB/cm and 1.0 dB/cm, respectively. This propagation loss of the e-skid waveguide is reasonable for compact devices, especially given that the cross-talks and bending losses are improved significantly (see Table I in the main text for comparison with other techniques).

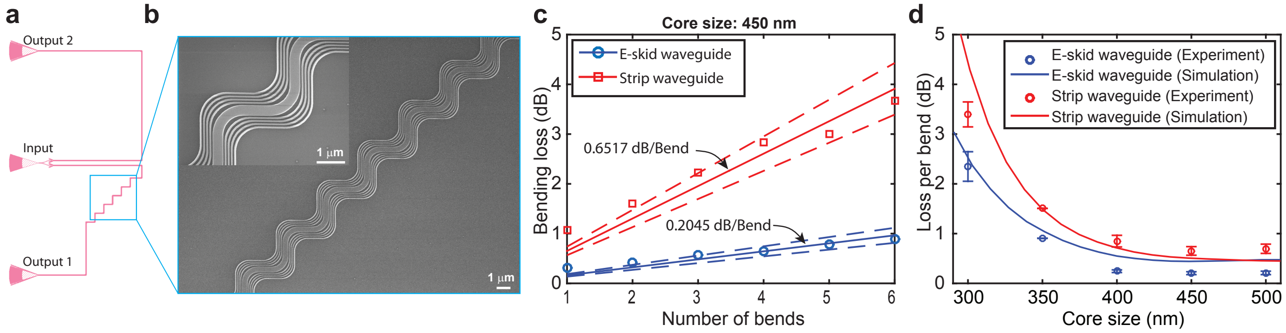

Supplementary Figure 9. **Bending loss. a,** Schematic representation of the experimental setup to measure the bending loss. Light is in-coupled through the middle grating coupler. It is divided into two branches of equal length but with a different number of bends. **b,** The top view SEM image of an e-skid waveguide with multiple bends. The inset shows a closer view. The bending radius is 1 μm and the other parameters of the waveguides are the same as the waveguides in Fig. 4 in the main text. **c,** The comparison of bending loss between e-skid and strip waveguides at telecommunication wavelength. The experimental points are fitted by a straight line. **d,** The comparison of simulated and measured bending loss in e-skid and strip waveguides versus the core size indicating a considerable reduction in the bending loss, specially when the core size is small.

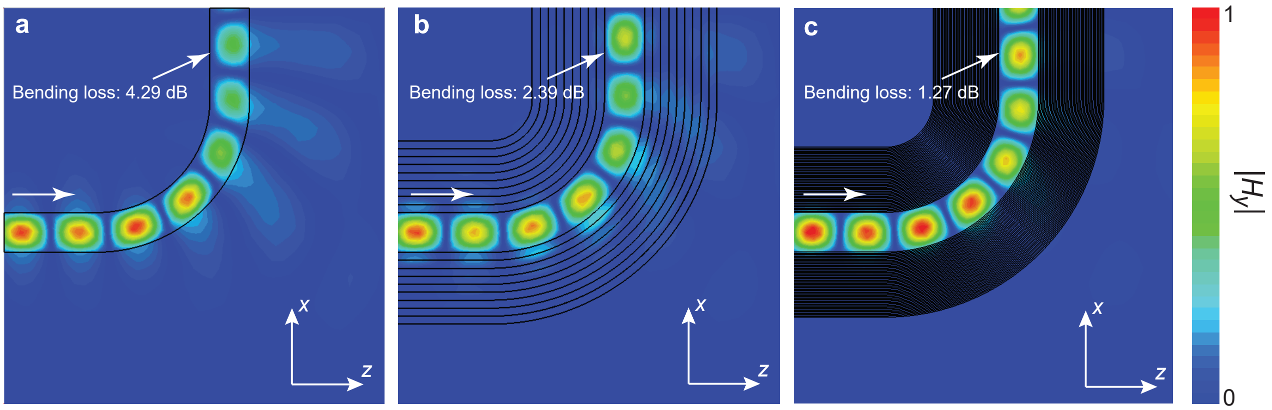

Supplementary Figure 10. **Full-wave simulation of the TE-like mode of curved waveguides.** Full-wave simulation results of the magnetic field of the TE-like mode of curved (**a**) strip waveguide, (**b**) e-skid waveguide with Λ=120 nm, and (**c**) e-skid waveguide with Λ=20 nm. The waveguide core size and bending radius are 300 nm and 1 μm, respectively. Other simulation parameters of the waveguides are the same as those in Supplementary Fig. 9. The waveguides are excited from the left side. The anisotropic cladding reduces the skin-depth in the cladding. Thus, the scattering at the bend is reduced in the e-skid waveguide case. The comparison of the field amplitude at the end of the waveguides clearly shows that the bending loss in e-skid waveguide is relatively lower. In the ideal case where the periodicity is deep subwavelength, the bending loss is considerably lower in e-skid waveguides.

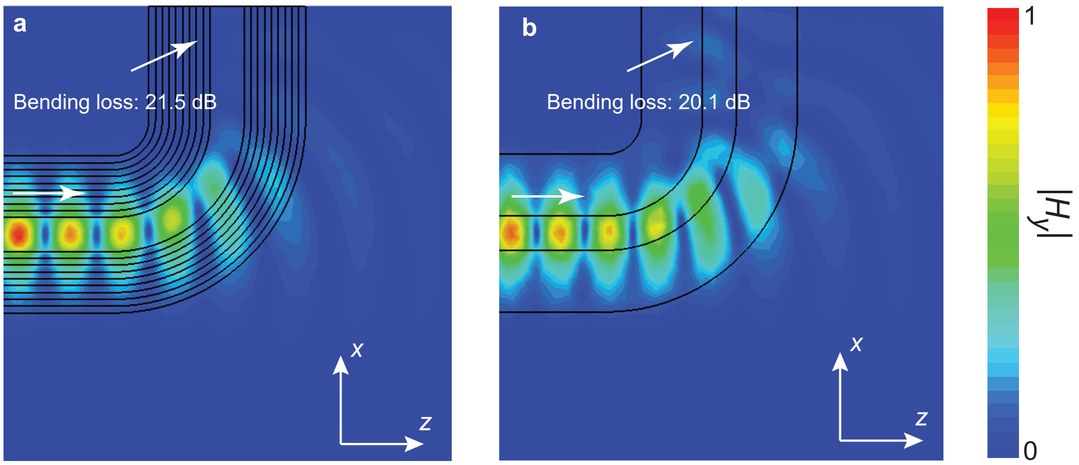

Supplementary Figure 11. **Full-wave simulation of the TM-mode of curved waveguides.** **a**, Full-wave simulation results of the magnetic field of the TM-like mode of curved e-skid waveguide with Λ=120 nm. Other simulation parameters of the waveguides are the same as those in Supplementary Fig. 10. **b**, The field profile of the same waveguide when the multilayer cladding is replaced by an isotropic cladding with the same permittivity as the effective permittivity of the multilayer metamaterial in the y direction. Since the electric field of the TM-like mode is polarized in the y direction and the electric field in the x direction is negligible, the anisotropy does not play any role in confinement. Thus, the TM-like mode is similar to that of an isotropic cladding waveguide. Note the TE-like mode probes the anisotropy of the multi-layer cladding and is fundamentally different.

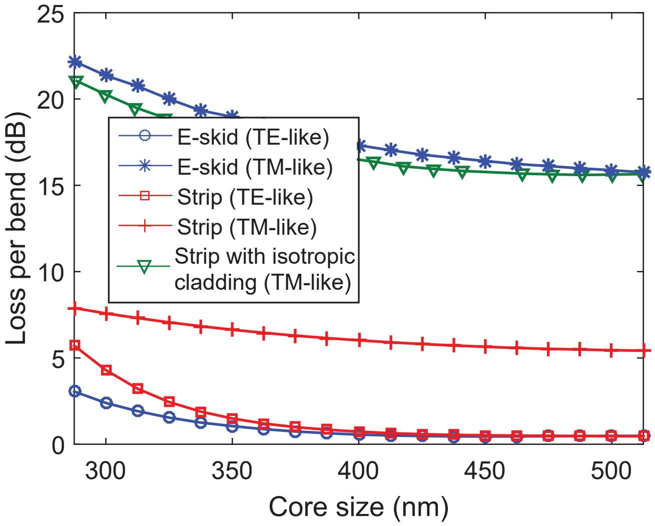

Supplementary Figure 12. **Comparison of the full-wave simulated bending loss in e-skid and strip waveguides for both TE-like and TM-like modes.** The structure is the same as in Supplementary Fig. 10. The bending loss for TE-like modes in e-skid waveguide is lower in comparison with that in strip waveguides because the multilayer cladding confines the light inside the core and reduces the skin depth in the cladding, especially when the core size is small and a considerable fraction of the total power is in the cladding. The green curve shows the bending loss for the TM-like mode when the metamaterial cladding is replaced by an isotropic cladding with the same size and the same permittivity as the effective permittivity of the multilayer metamaterial in the y direction. It is seen that the TM-like modes do not feel the anisotropy of the multilayer cladding. Thus, the anisotropic cladding cannot help to control the evanescent waves for TM-like modes. As a result, the bending loss for TM-like modes in e-skid waveguide is even worse than that in strip waveguides.

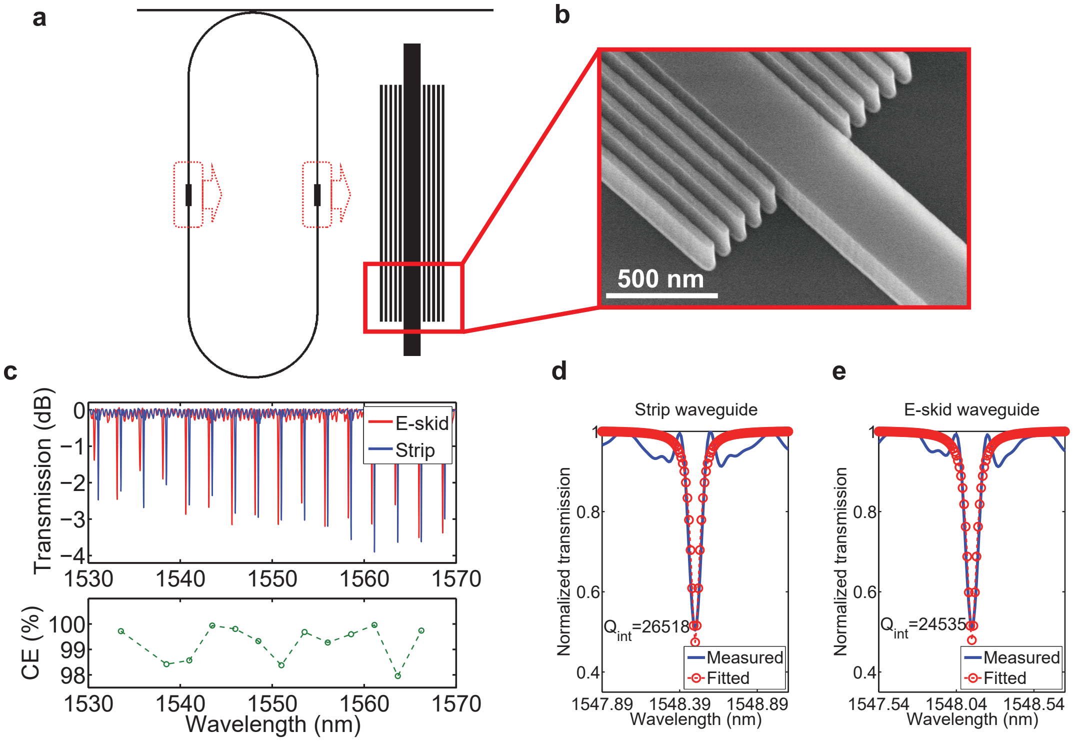

Supplementary Figure 13. **Mode conversion efficiency. a,** The schematic representation of the experiment set-up. **b,** SEM image of an e-skid to strip waveguide transition. **c,** Measured transmission spectra of the racetrack resonators without (blue) and with (red) the metamaterial claddings, and characterized mode conversion efficiency (CE). **d** & **e,** Fitted resonances: (**d**) without (strip waveguide) and (**e**), with (e-skid waveguide) the metamaterial claddings. The waveguide geometries are set to $h_0 = 220$ nm, $w_0 = 350$ nm, $w\rho = w(1-\rho) = 50$ nm, and $N = 5$.

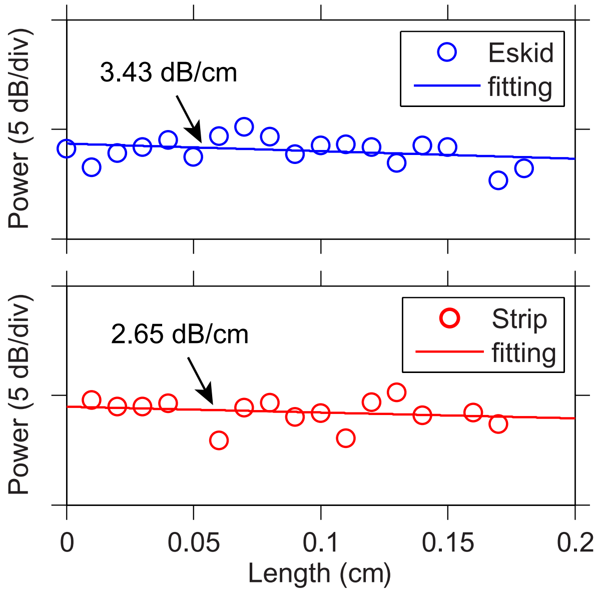

Supplementary Figure 14. **Propagation losses in e-skid and strip waveguides.** The circles represent normalized transmission through straight e-skid (blue) and strip (red) waveguides at $\lambda = 1550$ nm. The length of the waveguides is varied from 0 to 1.8 mm to characterize the propagations loss (fitting curves). The propagation loss in e-skid and strip waveguide is 3.43 and 2.65 dB/cm at $\lambda = 1550$ nm, and the average losses for e-skid and strip waveguides for wavelengths between 1540 nm to 1560 nm are 3.67 dB/cm and 1.84 dB/cm, respectively, with a standard deviation of 1.0 dB/cm and 1.4 dB/cm, respectively. The other waveguide parameters are $w_0 = 450$ nm, $h_0 = 220$ nm, $w_\rho = w_{(1-\rho)} = 50$ nm, and $N = 5$. This clearly shows that e-skid waveguides have comparable losses to standard strip waveguides opening the path for practical applications.

SUPPLEMENTARY REFERENCES

[1] D. Dai, Y. Shi, and S. He, Applied Optics **46**, 1126 (2007).

[2] CST Microwave Studio, http://www.cst.com .

[3] L. Chrostowski and M. Hochberg, *Silicon Photonics Design: From Devices to Systems* (Cambridge University Press, 2015).

[4] K. Han, S. Kim, J. Wirth, M. Teng, Y. Xuan, B. Niu, and M. Qi, Optics Express **24**, 6532 (2016).

[5] S. Xiao, M. H. Khan, H. Shen, and M. Qi, Optics Express **15**, 10553 (2007).
